# Simulating Elastoplastic and Anisotropic Behavior in Thermoplastic Additively Manufactured Components: An Application-Oriented Modeling Approach

**DOI:** 10.3390/polym16091234

**Published:** 2024-04-28

**Authors:** Fabian Ferrano, Miranda Fateri, Markus Merkel, Jan Hertel

**Affiliations:** Faculty Mechanical Engineering & Materials Science, Aalen University, Beethovenstr. 1, 73430 Aalen, Germany; miranda.fateri@hs-aalen.de (M.F.); markus.merkel@hs-aalen.de (M.M.); hertel_jan@gmx.de (J.H.)

**Keywords:** 3D printing, additive manufacturing, fused filament fabrication, FEM simulation, coupled simulation, elastoplastic material, anisotropy, fracture behavior

## Abstract

This paper presents a comprehensive approach aimed at developing a coupled process-structure simulation that integrates anisotropic and elastoplastic material behavior for plastic components manufactured through Fused Filament Fabrication (FFF) 3D printing. The simulation incorporates material orientation considerations, linking the process simulation with structural simulation. Subsequently, stress and strain values from the simulations are compared with the test results. Moreover, the fracture behavior of components manufactured in this way is also taken into account in relation to material orientation. The executed simulations have yielded successful outcomes, affirming the efficacy of the anisotropic and elastoplastic simulation across all strand orientations. Special attention is paid to the application of the method. Here, the simulation method introduced in this contribution with the approaches for describing the material behavior under mechanical load can be used in the future in the dimensioning of FFF manufactured plastic components to predict the deformation behavior and failure, especially under consideration of a well economic and efficient virtual product development.

## 1. Introduction

Additively manufactured plastic components are gaining growing significance [1], both in personal applications and across diverse industries [2]. The primary reason behind this trend is the infinite possibilities offered by additive manufacturing, which presents industries with novel avenues to conceptualize their products. In recent decades, several AM techniques have been developed for plastic applications. These AM techniques primarily include Fused Filament Fabrication (FFF, trademarked as FDM) [3,4,5] stereolithography (SLA) [6,7,8], selective laser sintering (SLS) [9,10,11] and PolyJet modeling (PJM) printing [11,12,13,14].

Recently, FFF, a subset of Material Extrusion (MEX) processes, has gained significant attention in technical fields [14]. Consequently, the assessment of the mechanical behavior of FFF-produced items under stress conditions is becoming increasingly crucial. Subsequently, it is also becoming increasingly important to be able to evaluate the mechanical behavior of FFF products under stress conditions. Among the tested materials for FFF, Polylactide (PLA) as a biodegradable polymer [15,16,17] and Acrylonitrile Butadiene Styrene (ABS) as a non-biodegradable polymer [16,18,19] have been widely investigated with regard to their mechanical properties. Polylactic acid (PLA) is utilized in additive manufacturing technologies for its deformation property and cost-effectiveness [15,20,21]. Components made of ABS have similarly excellent mechanical properties, with a higher elongation at break and a tendency towards lower stress at break [22,23,24].

As reported, components manufactured using the FFF process exhibit distinctive mechanical behavior due to their directional layer-by-layer structure [1]. The inherent anisotropic and elastoplastic material responses present challenges in their computational representation within Finite Element Method (FEM) simulations [25]. Current Finite Element Method (FEM) software systems do not currently facilitate the integration of the printed material’s orientation into the FEM calculation. Nevertheless, the alignment of the material holds significant importance in shaping the strength attributes of the printed component [1]. Hence, it is crucial to account for material orientation in simulations to ensure realistic outcomes. The mechanical characteristics of materials produced through FFF, incorporating different raster angles along the thickness direction, can be assessed using methodologies established for laminates [26,27,28] which mainly draw inspiration from various techniques used in understanding fiber composite materials, where a highly rigid fiber interacts with a polymer matrix [29].

Also, it is possible to explore the potential for describing anisotropic material behavior through meso-macro modeling [30], revealing localized variations in stiffness and strength. By applying homogenization methods [31] at the meso level, it is possible to pinpoint parameters that depict local anisotropy and how the material behaves when experiencing deformation at the macro level [29,32,33]. This method is especially employed when simulating short-fiber-reinforced thermoplastic materials that are processed by injection molding [34,35].

The aim of the present study is to develop a coupling of the process and structure simulation by using meso–macro–approach. Through the coupling, the orientation of the material in the process simulation should be considered in the FEM calculation. In addition to this, isotropic and elastoplastic FEM calculations are compared to this method in order to evaluate the process-structure coupled simulation with respect to the simulation characterization effort and the goodness of results. Moreover, an anisotropic and elastoplastic material model has to be created, which represents the material behavior for the corresponding orientation of the material. With the coupled simulation, it should be possible to simulate the mechanical material behavior for arbitrary filling, close to manufactured specimens.

The results of the coupled simulation are then compared with test results using components with a typical 45°–135°-layer structure. Subsequently, the simulation model created is to be tested for different variants of the tension specimen. In particular, the use of the process-structure coupled simulation in a product creation process is evaluated and a recommendation is made accordingly.

The local failure is described in the context of material orientation. Suitable limit values were investigated by Ferrano et al. [36] and Sabik et al. [37] for components manufactured using the FFF process, but without taking anisotropy into account.

As part of this research, a coupled process-structure simulation that considers the anisotropic and elastoplastic mechanical material behavior of additively manufactured components was developed. The results show that the simulation accurately reproduces the material behavior, including directionality and prediction of local failure, providing valuable insights for the optimization of designs and further development of additive manufacturing applications.

## 2. Quasistatic Material Behavior

**Figure 1:**
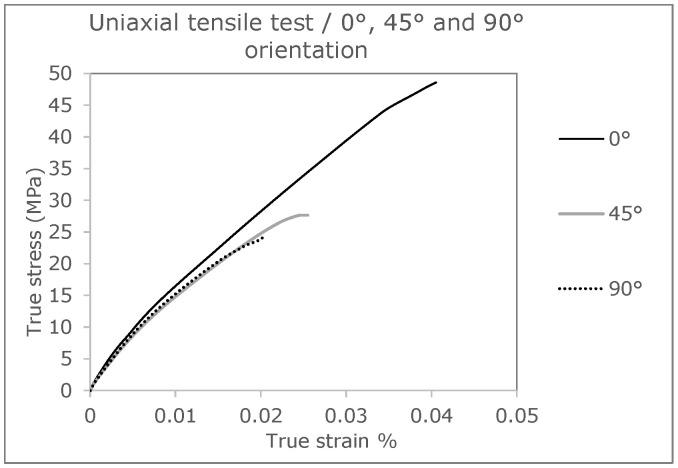
True stress-strain curves with different strand orientations (0°, 45° and 90°) [36].

**Figure 2:**
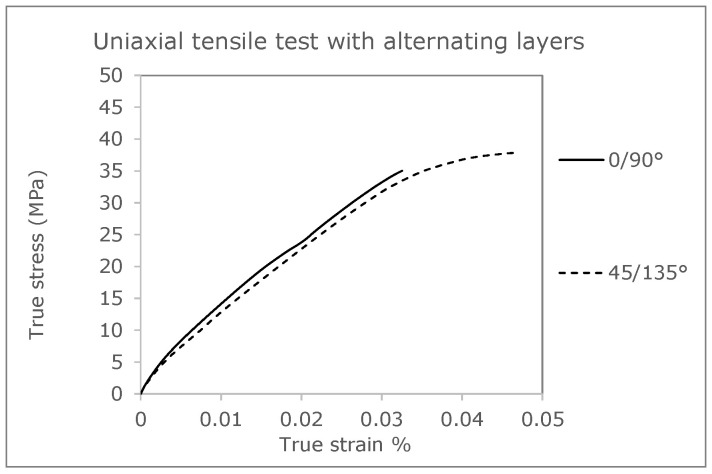
True stress-strain curves with cross-layered structured 0°/90° and 45°/135° [36].

Looking at the results of the samples with an orientation of 90°, as shown in Figure 1, the influence of the strand orientation becomes obvious. In this variant, component failure occurs earliest. This is due to the interfaces of the individual strands, which run parallel to the tensile stress. As a result, an enormous concentration of stress can form between the strand connections, causing the samples to fail at this point. Such failure behavior is explained in more detail in Section 3.2 on crack development in FFF components. Compared to the test specimens with a strand orientation of 0°/90° (see Figure 2) in the bending test, the modification of the specimens for the tensile test leads to an increase in the mechanical properties. The changing structure of the strands at 0° and 90° thus represents an optimization of the 0° specimen. The strands in the direction of the load at 0° can compensate for the weak points in a tensile load and lead to an improvement in the mechanical properties.

To validate the coupled simulation, results from the investigations of Ferrano et al. [36] will be used later. It should be mentioned that all experimental data (stress-strain curves) are taken from this publication for this comparison. Tensile tests of the specimens with an alternating layering of 0°/90° and 45/135° are used to evaluate an application-related transverse arrangement of the layers frequently used for technical components.

**Figure 3:**
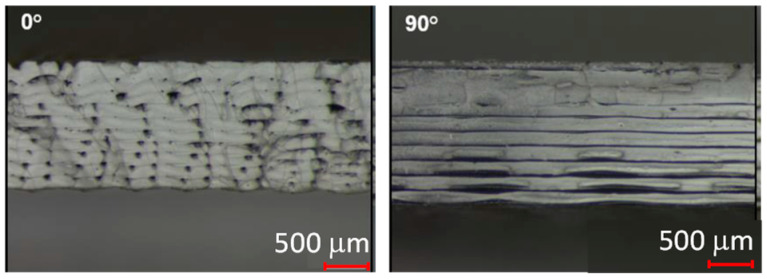
Porosity of tensile test rods in the 0° and 90° deflection direction.

Directional dependence is affected by the adhesion between the individual strands and the layers. In addition, porosity can be seen when looking at the fracture surfaces of the tensile test specimens with different strand deposition directions. In this study, an average porosity of 6.9% can be determined. This is accomplished by measuring the samples and the resulting volume. The porosity can be determined via the mass and the comparison of the density of the polymer. Figure 3 shows the porosity of tensile test bars in the 0° and 90° deflection directions on fracture surfaces.

## 3. Simulation Methods

The mechanical material behavior under the quasi-static load described in Section 2 motivates the use of an elastoplastic material model in FEM analyses. In addition, the local strand direction averaged over all possible directions can be taken into account in a simplified manner. This corresponds to an isotropic elastoplastic simulation of the quasi-static material behavior. This approach is in contrast to the anisotropic elastoplastic FEM analysis, in which the local material behavior can be mapped. This has two advantages: the deformation behavior can be described in detail and close to the true behavior, and it is also possible to detect failures in the individual layers.

### 3.1. Structural Simulation/Isotropic and Elastoplastic Approach

**Figure 4:**
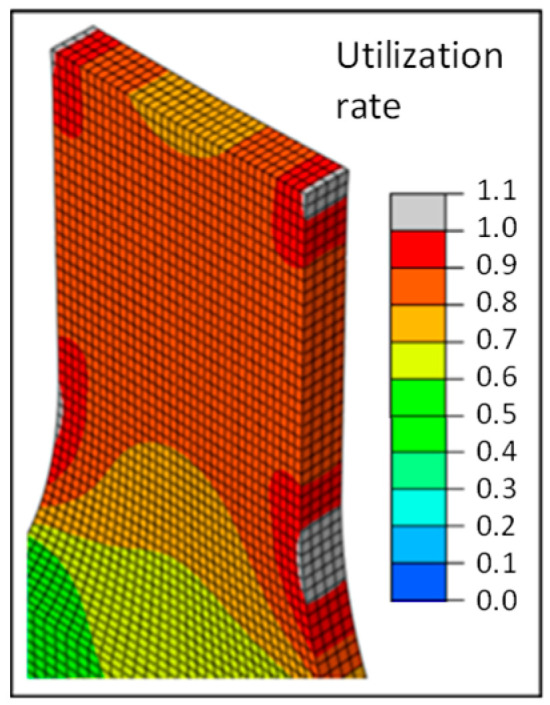
Local failure of the isotropic FEM model.

For the isotropic and elastoplastic FEM analysis, the FEM program Abaqus^®^ is used. Here, the tensile specimen to be simulated is first modeled and the boundary conditions are created according to a tensile test. For this purpose, the geometry of the Becker tension specimen [2] is used. Square quadrilateral elements with an edge length of 0.5 mm each are chosen to discretize the volume. This results in an only slightly distorted hexahedral mesh for the complete tension bar (see Figure 4). Thus, four elements, respectively, 8 integration points result over the 2 mm tension specimen thickness. This mesh size is the result of a sensitivity analysis, with which it can be ruled out that the mesh size has an influence on the calculated stress and strain result.

The isotropic FEM analysis does not consider the strand orientation of the component in the simulation, resulting in the same stress distribution for each analysis. Manual adjustment of the material parameters in the FEM model is not practical, especially if anisotropies are to be described locally and in detail by means of a suitable model.

The use of an averaged or reduced stress-strain curve to describe the deformation behavior and failure is applied in the field of short glass fiber-reinforced plastics [38]. In particular, the procedure according to Lopez et al. [39] is recommended here, where the stresses from the tensile test of a 0° specimen are weighted with the factor 3/8 and the 90° tensile stress with 5/8. Here, too, there are local anisotropies that can be attributed to the manufacturing process. This procedure has also proven effective for components manufactured using the FFF process [36,40].

The elastoplastic material behavior is determined by means of test results of the Poisson’s ratio, the modulus of elasticity, and the course of the true stress and plastic strain. The von Mises yield criterion (J2—plasticity) F is used to describe plastic deformation, with which the deformation behavior of a tensile loaded polymers can be well described [27], and it is based on the following equation:

(1) $$F=\sigma_{eq,vM}-\sigma_{F}\sqrt{\frac{1}{2}\left[{\left(\sigma_{11}-\sigma_{22}\right)}^{2}+{\left(\sigma_{22}-\sigma_{33}\right)}^{2}+{\left(\sigma_{33}-\sigma_{11}\right)}^{2}\right]+3\left(\sigma_{12}^{2}+\sigma_{23}^{2}+\sigma_{31}^{2}\right)}-\sigma_{F}=0$$

Considering hardening, the equation for the flow condition *F* is as follows, using a strain hardening function $R\left(\varepsilon^{p}\right)$.

(2) $$F=\sigma_{eq,vM}-\sigma_{F}-R\left(\varepsilon^{p}\right)$$

The plastic deformation behavior of semi-crystalline polymer materials is coupled with strain hardening [41]. The failure is determined via a utilization rate, following the studies of Ferrano et al. [36], according to the following equation:

(3) $$A_{LE}<\frac{\epsilon_{LE}}{\varepsilon_{max}};\epsilon_{LE}=max\left|\varepsilon_{1},\varepsilon_{2},\;\varepsilon_{3}\right|$$

Here, the maximum logarithmic strain ($\epsilon_{LE}$) is used for the failure criterion. As a result of the unchanged stress distribution in the isotropic FEM analysis of the different variants, the same fracture behavior is obtained in each case.

**Figure 5:**
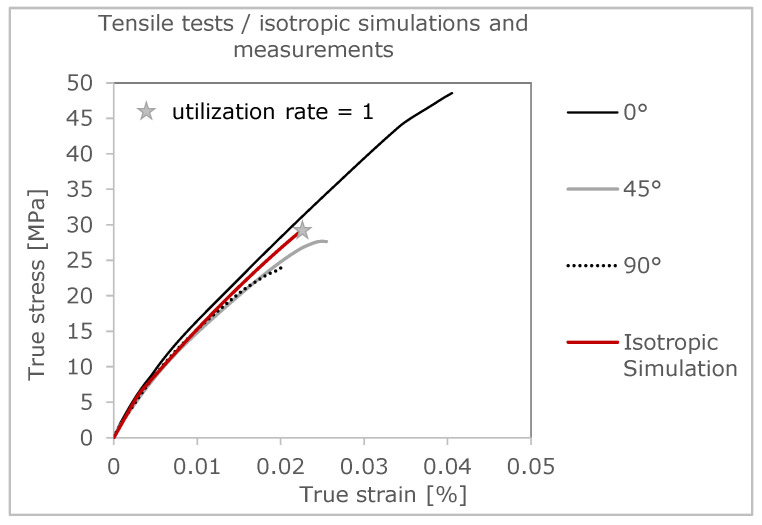
Deformation behavior of the isotropic FEM model.

In Figure 4 and Figure 5, simulation results are summarized and compared to test results. The deformation behavior can be described in an adequate way, whereas no direction dependency can be considered. The disadvantage of the isotropic FEA is obvious when focusing on predicting the damage. There is no correlation to test results, especially for the 0°-strand direction. The local failure of the isotropic FEM model is shown in gray in Figure 4. It can be seen that the local failure in the isotropic FEM analysis starts at the beginning of the radii on the outer wall, since this is the geometry-related weak point of the component.

### 3.2. Structural Simulation/Anisotropic and Elastoplastic Approach

**Figure 6:**
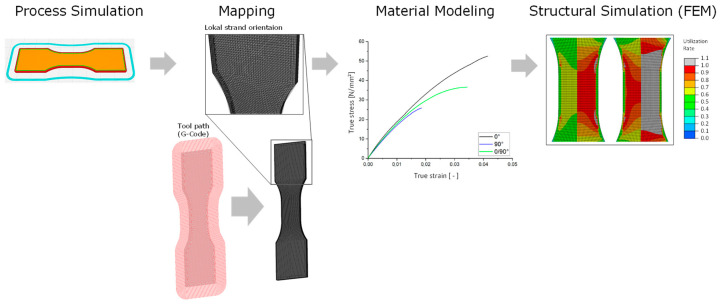
Steps of a process and structured coupled simulation considering local manufacturing direction.

This is followed by the presentation of the quasi-static FEM analysis of the anisotropic and elastoplastic material behavior created from the directional tensile test. Here, a coupled process-structure simulation is performed. The aim is to characterize a material model which can be applied for an arbitrary strand orientation based on the tension specimen [42] with 0°, 90° orientation and which generates a realistic prediction of the mechanical behavior for FFF manufactured components as well as for reinforced thermoplastics in general [33]. After realizing process simulation and creating a basic FEM model, local strand orientation is mapped on FEM mesh, the material model has to be fitted, followed by coupling of local strand orientation and material behavior for structural simulation. The basic procedure is shown in Figure 6.

As in the isotropic simulation, the structural model is modeled in the FEM software Abaqus^®^. This software is later used to perform the coupled FEM analysis and to read out the results of the anisotropic and elastoplastic FEM analysis. In addition to this, the Digimat^®^ 2022.2 software is used to transfer the information of the local strand orientation to the structural mesh (FEM mesh), which is also referred to as mapping.

First, the machine code (G-code) of the part to be manufactured is imported, followed by the structural mesh. With the help of the G-code, it is now possible to determine a direction vector for the coordinate of each node of the structure mesh locally. This information is then used in a subsequent step to describe an anisotropic material behavior (see Section 3.2.2 Material model). Finally, the information of the direction of the anisotropy is transformed into a direction dependence of the material behavior and the coupled process-structure simulation is started, integrating the previously generated files.

#### 3.2.1. Process Simulation

**Figure 7:**
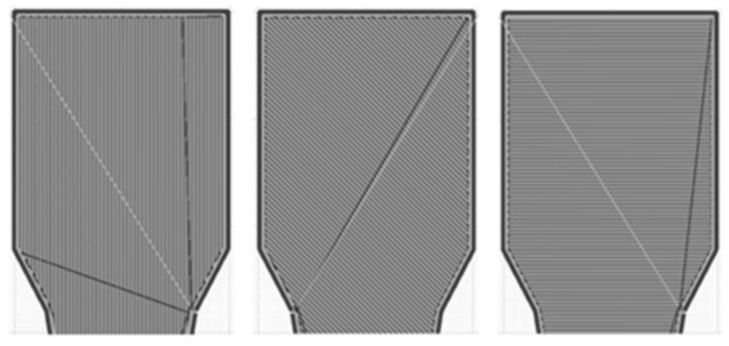
Top view of strand orientation in slicing software: 0°, 45°, and 90°.

The Ultimaker Cura^®^ 4.8 software is applied for the process simulation. This is used to determine the layer thickness and strand orientations (Figure 7) relevant for the anisotropic FEM analysis. As for the tensile test, the three variants, 0°, 45°, and 90° strand orientation, are generated as filling of the tensile specimens. The two outer lines, which limit the filling of the tensile specimen, remain the same for each variant. There are no edge layers covering the infill from the top or bottom side. These data are stored as G-code, which is relevant for the coupled process-structure simulation.

**Figure 8:**
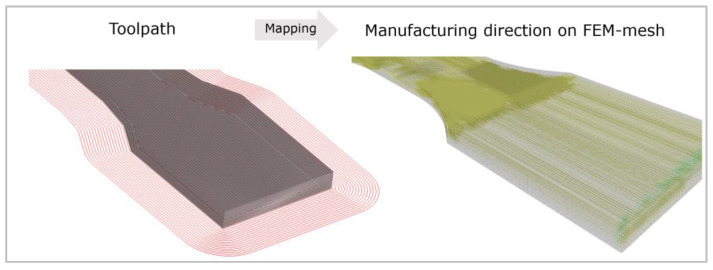
Mapping.

In the next step, the generated data of the process simulation are transferred to the FEM model. The STL file is selected as the donor mesh. The G-code with the desired strand orientation, the so-called mapping, is loaded for this file. The input file is selected as the receiving mesh. Then the alignment of the receiving geometry to the donating geometry is completed because both need exactly the same alignment to position all information of the G-code at the right place. Here, the orientation tensors of the nodes of the process mesh are placed on the nearest nodes of the structural mesh. Thus, an XML file is generated with the orientations at the respective points. Figure 8 shows the mapping of the process simulation, of a tension specimen with 0° filling, onto an FEM model.

#### 3.2.2. Two-Step Homogenization

In order to describe the specific material behavior, this is created by a self-programmed material model in Fortran, which is implemented with the help of a so-called UMAT (User Material) for Abaqus. First, the true stress and strain values of the three curves (0°, 45°, and 90° strand orientation) from previous tensile tests are considered to fit the parameters for this material model. The material behavior of raw (solid) PLA is described as elastoplastic and isotropic. The von Mises plasticity is used for the plastic behavior. This is referred to as J2 plasticity model. The preliminary stress-strain curve of the initial material generated from the inputs corresponds to the 0°direction (see Figure 1). In order to be able to specify the microstructure of the component, a porosity measurement of a 0° test specimen is carried out. From this measurement, it is found that the tensile test bar contains a defect volume of 6.89%, due to fabrication by the FFF process. This finding is important for coupled process-structure simulation, since the size of the void has a huge impact on the anisotropy of the part [1].

**Figure 9:**
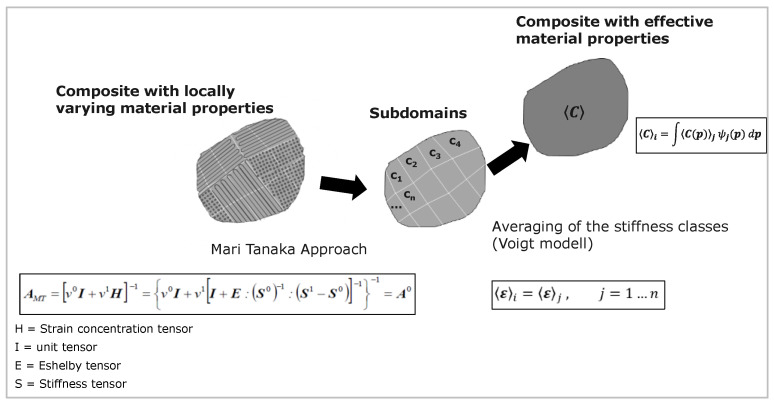
Two-Step Homogenization Scheme.

It is determined that the material model is created for the purpose of mechanical analysis. The model used in this work consists of two homogenization steps and is based on the approaches of Mori-Tanaka [29,35] and Reuss [18] as figured out in Figure 9.

The latter is based on the assumption that a constant stress field prevails in the volume domain. The Mori–Tanaka model is based on the model of Eshelby [31]. He developed a theory about the amplification effect of ellipsoidal inclusions (principle of equivalent influences). Eshelby’s model allows for the transformation of the properties of the inclusion to the surrounding medium with the help of a fictitious self-expansion of the inclusion [30]. Mori–Tanaka extended this assumption that there is a nearly constant field between the inclusions, namely the mean strain of solid PLA [32]. They mentioned that this assumption of inclusion can exist in a matrix of the same material, and it motivates using FFF fabricated components. In contrast to [13], where the assumption is based on the laminate theory, a single-material continuum can be used as a basis in micromechanics. After the initial material has been described, the next point is the microstructure. This item defines the type and ratio of the material. For the additively manufactured component, the lattice microstructure is selected. Once the starting material and the microstructure are described, the determination of the material failure follows. Here, the failure behavior of the material is described. In order to be able to describe the quasi-static failure of the component, the Tsai–Hill criterion [43] is applied:

(4) $$f_{i}=\sqrt{F_{i}(\sigma )},\;\mathrm{with}:$$

(5) $$F_{A}(\sigma )=\frac{\sigma_{11}^{2}}{X^{2}}-\frac{\sigma_{11}\left(\sigma_{22}+\sigma_{33}\right)}{X^{2}}+\frac{\sigma_{22}^{2}+\sigma_{33}^{2}}{Y^{2}}+\left(\frac{1}{X^{2}}-\frac{2}{Y^{2}}\right)\sigma_{22}\sigma_{33}+\frac{\sigma_{12}^{2}+\sigma_{13}^{2}}{S^{2}}+\left(\frac{4}{Y^{2}}-\frac{1}{X^{2}}\right)\sigma_{23}^{2}$$

Failure occurs when the ratio of a principal stress or shear stress and a directional limit value is greater than or equal to 1. The corresponding values for the material considered in this study are shown in Table 1. X corresponds to the limit stress for the 1st principal stress, Y to the 2nd principal stress and S to the limit value for the maximum shear stress. Using the Tsai-Hill criterion which can be graphically displayed in subsequent FEM calculations to evaluate the quasi-static failure of the component. In connection with the isotropic simulation, a degree of utilization can also be introduced here.

**Figure 10:**
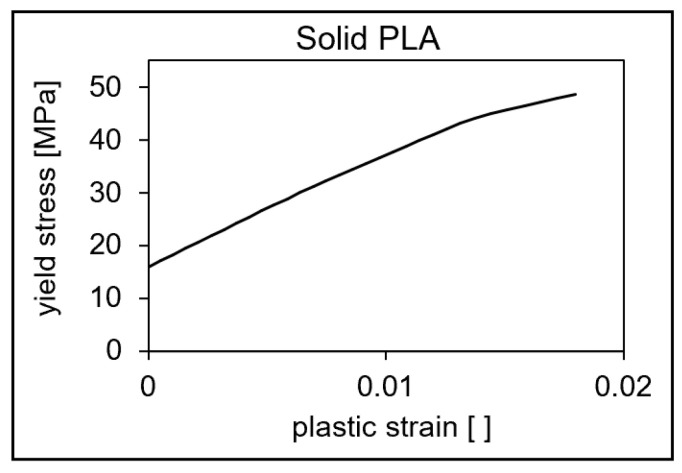
Plastic strain–true stress curve for pure PLA.

The adjustment of the parameters for the description of the anisotropy is carried out with the help of the iSIGHT^®^ 2022 Software by adjusting the parameters shown in Table 2 in the simulation model using a 0° and 90° oriented sample. As a target variable, the stress-strain curves from the simulation and test are compared and the stresses are compared to a defined strain in each case and the curves are approximated by adjusting the input parameters using the method of least squares. Here, scalar variables for the solid PLA material as well as variables of the inclusion are varied. In addition, the elastoplastic material behavior of the pure PLA is adapted by means of the plastic strain–true stress curve so that the non-linear, plastic behavior can be reproduced with sufficient accuracy (see Figure 10).

## 4. Simulation Results/Deformation Behavior

In this chapter, the stress-strain curves obtained from the coupled process-structure simulation are compared with those obtained from the test. Three simulations were performed for this purpose. For each simulation, the created anisotropic and elastoplastic material model is used.

**Figure 11:**
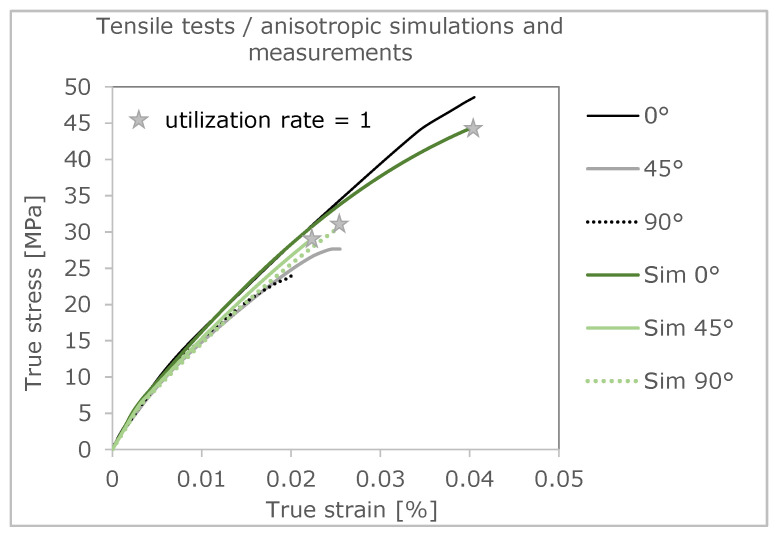
Comparison of the true stress-strain curves of tensile test and simulation.

The three simulations give out different stress-strain curves and failure evaluations as a result. This is positive that the differences in material orientation also show an influence. In addition, it is worth highlighting that the failure of the real tension members is very accurately represented by the simulation using the Tsai–Hill failure criterion [3]. In the following figure (Figure 11), the curves of the simulation are compared with the curves of the real tensile test.

**Figure 12:**
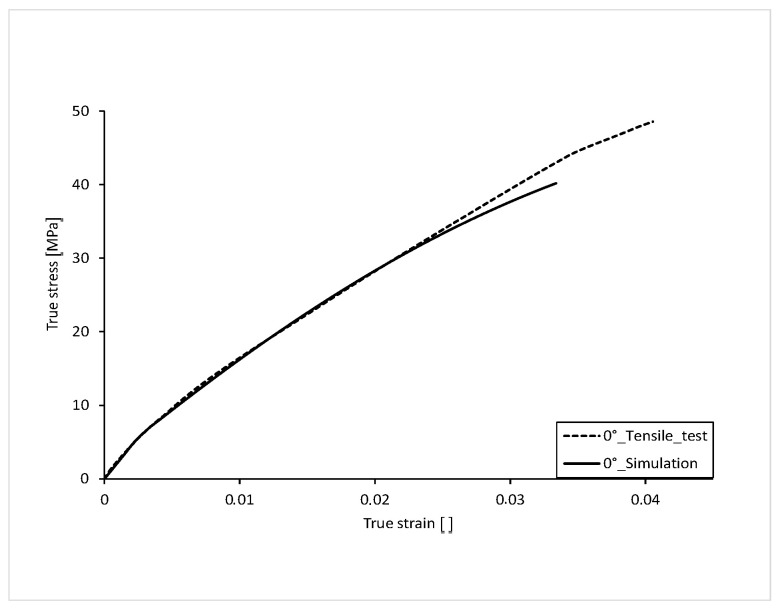
Comparison of true stress-strain curve 0°.

The stress-strain curve with 0° orientation of the filling in the tension bar is shown in Figure 12. For comparison, the curve from the tensile test is also shown. With a fracture stress of 40.19 MPa, the stress-strain curve of the 0° simulation is about 16% lower than that of the test. The elongation at break, with a value of 0.0338, also differs by approximately 16% than that of the test. This deviation in ultimate strain and stress results from the fact that in the tensile test, even after minimal local failure, the tensile bar is still capable of loading for a short time and does not break immediately. In the simulation, the curve is terminated at local failure. Local failure is described in more detail in the following chapter.

**Figure 13:**
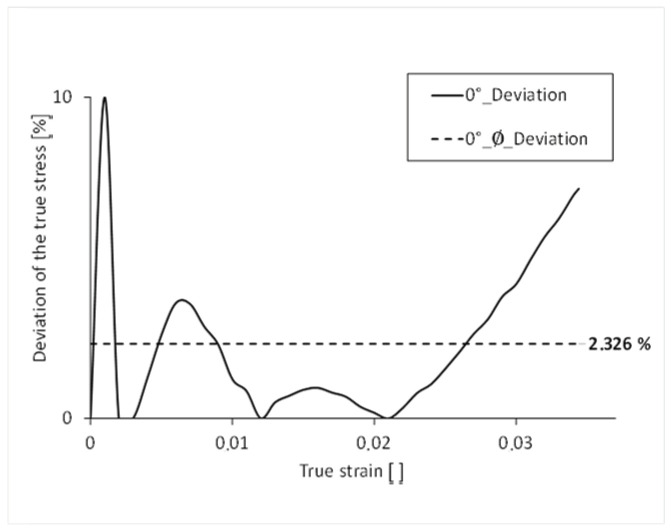
Percentage deviation of the stress over the course of the strain 0°.

To be able to compare the two curves over their course, the percentage difference of the true stress is mapped over the course of the true strain. The simulated stress-strain curve has an average percentage deviation of the stress of 2.392% over its complete course compared to that of the test, and thus a realistic result of the simulation (Figure 13). The largest percentage deviation of stress can be seen at the beginning of the curve. This is due to the fact that the elastic range of the real tensile test is not exactly linear.

**Figure 14:**
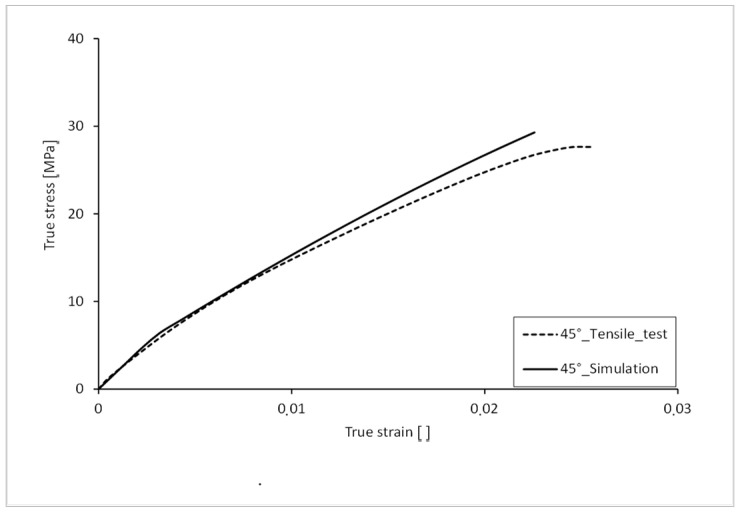
Comparison of true stress-strain curve 45°.

The stress-strain curve with 45° orientation of the filling in the tension bar is shown in Figure 14. For comparison, the curve from the real tensile test is also shown. A deviation can be seen in the elastic range up to approx. 6 MPa. The Young’s modulus of the simulated curve is slightly higher. The plastic strain hardening is more arc-shaped in the test values, which is why the curve of the simulation deviates from the test values here. With a fracture stress of 29.30 MPa, the simulation curve is 6.01% lower than that of the test. The elongation at break, with a value of 0.022, is 11% lower than that of the test.

**Figure 15:**
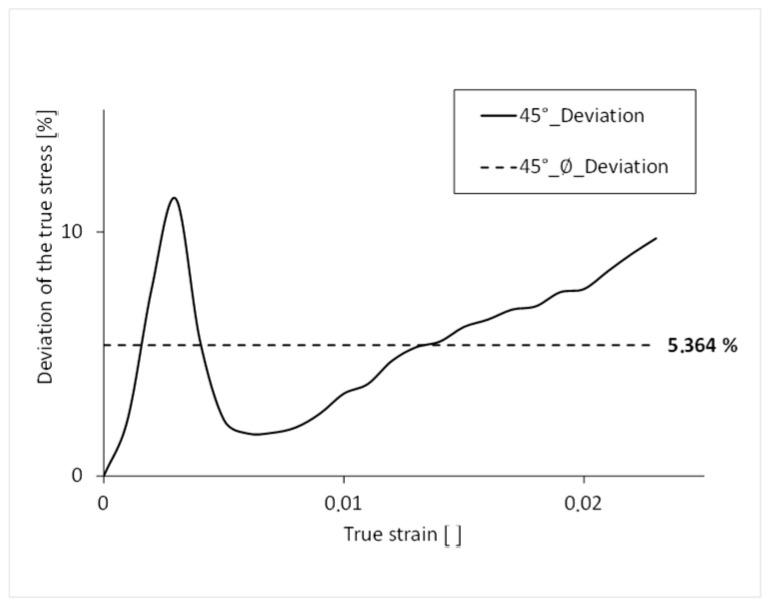
Percentage deviation of the stress over the course of the strain 45°.

The simulated stress-strain curve has an average percentage deviation of 5.4% over its complete course compared to that of the real test and thus a good result (Figure 15). The largest percentage deviation of the stress can be seen at the yield point.

**Figure 16:**
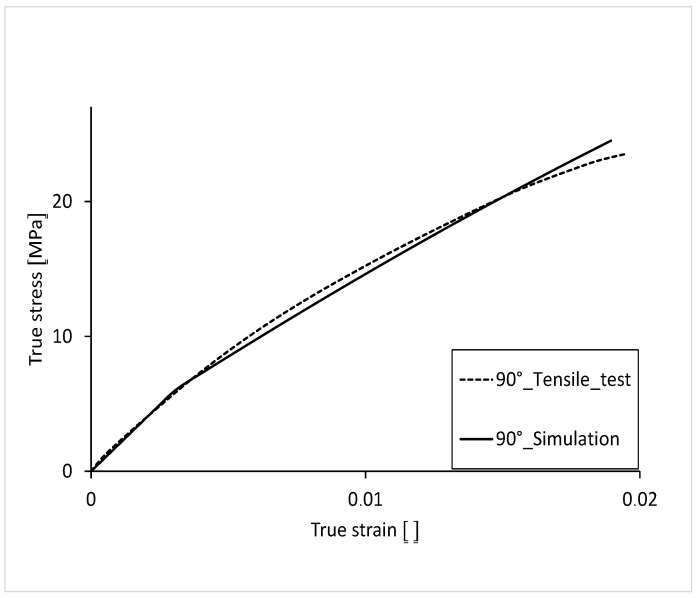
Comparison of true stress-strain curve 90°.

The stress-strain curve, with 90° orientation of the filling in the tension bar, is shown in Figure 16. For comparison, the curve from the tensile test is also shown. As with the 45° variant, the plastic curve is not as arc-shaped as that of the test. With a fracture stress of 24.50 MPa, the simulation curve is 1.7% lower than that of the test. The elongation to failure, with a value of 0.0189, is 6.4% lower than that of the test.

**Figure 17:**
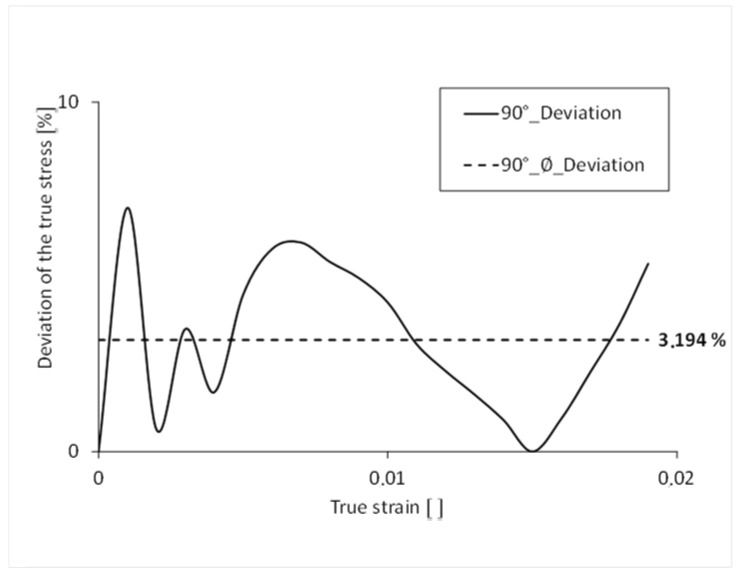
Percentage deviation of the stress over the course of the strain 90°.

The simulated stress-strain curve has an average percentage deviation of 3.2% over its complete course (Figure 17). The largest percentage deviation of stress can be seen at the beginning of the curve. The simulation results reflect the results of the real tensile test very well. The following table (Table 3) shows the average deviation of the true stress over the course of the true strain.

In the following tables (Table 4 and Table 5), the fracture stress and strain of the tensile test are compared with those of the simulation.

In summary, both anisotropy and failure can be well described using this approach. The 0° direction has the largest differences in both stress and strain, whereas at a 90° layering direction, the differences are significantly smaller. Last but not least, the comparisons of the deformation behavior in Figure 12, Figure 14 and Figure 16 enable an evaluation of the results in relation to the level of load. A sufficiently accurate prediction of the mechanical properties can also be summarized.

**Figure 18:**
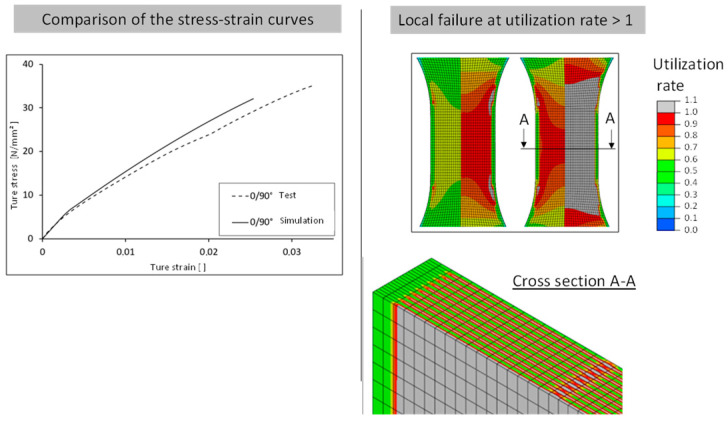
Comparison of deformation behavior and local failure.

A further validation, in addition to the 45° specimen, which does not serve as an input variable for the adjustment of the material model, is to be carried out by comparing cross-laid tensile test bars. Figure 18 shows the layered structure of a tension rod with 0°/90° direction of layering. Here, the failure of the individual layers is clearly visible, and, for example, the overall failure of the tension rod can be evaluated and compared with a test result. Furthermore, the failure behavior can be attributed to the different strength (breaking stress) as is taken into account using the Tsai–Hill criterion. Areas loaded at 90° to the placement direction therefore fail faster than areas in the main direction, which is related to the typical anisotropic behavior of the investigated material and the manufacturing process.

### Simulation Results/Fracture Behavior

This is followed by a comparison of the fracture behavior of the real tension specimens with those of the anisotropic and elastoplastic simulation. Here, the utilization rate is considered in the simulation. This value is calculated using the Tsai–Hill criterion [3]. The simulated local failure indicates a crack of the component.

**Figure 19:**
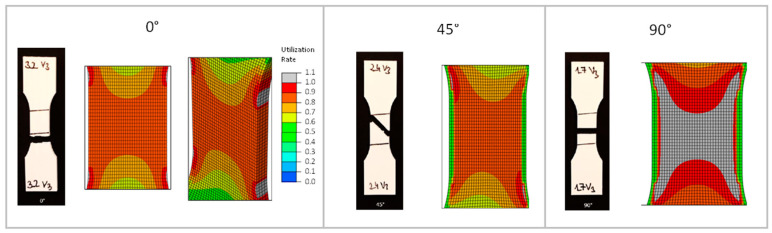
Comparison of the fracture behavior of the tension specimens and simulation.

In the following illustrations (Figure 18 and Figure 19), the fracture behavior of the 0° test and the simulated anisotropic FEM model with 0° strand orientation is shown on the left. The local failure of the FEM model is shown by the four gray zones at the beginning of the radius. The tensile member of the test also exhibits a fracture at the beginning of the radius. Furthermore, it can be seen that in the FEM model, the local failure starts at the outer wall. This results from the fact that all strands, including both outer lines, have the same orientation in the constricted part of the tension bar.

The failure behavior of the 45° test and the simulated anisotropic FEM model with 45° strand orientation is shown in the center. Two gray zones of local failure can be seen. As with the 0° variant, the zones are located at the beginning of the radius. These two zones are not horizontal, but diagonal to each other. Connected, they represent the diagonal fracture behavior of the tension specimen from the test. Thus, the simulation also shows a fracture behavior along the strand orientation. Unlike the 0° variant, the local failure does not occur at the outer wall, but at the point where the 45° infill is connected to the outer lines. The 0° outer lines have a higher strength than the 45° filling. The same can be seen with the 90° variant. The zones of local failure start at the beginning of the radius in all four corners and collect in the center of the tension bar.

## 5. Conclusions and Discussion

The increasing significance of additively manufactured plastic components in various applications necessitates a thorough understanding of their mechanical behavior, especially under stress conditions. Fused Filament Fabrication (FFF), a prominent additive manufacturing technique, poses unique challenges due to its directional layer-by-layer structure, resulting in anisotropic and elastoplastic material responses. The goal of creating a coupled process-structure simulation taking anisotropy into account was successfully achieved. The result of this publication is a coupled simulation which, considering the directional dependence of the anisotropic and elastoplastic material, represents a realistic representation of the material behavior. The comparisons with the simple orientations (0°, 45° and 90°) of the material resulted in a realistic representation by the simulation. The simulations of the 0/90° and 45/135° tension bars proved that combinations of the directions can also be represented in the simulation.

The representation of the material behavior of the individual layers showed the advantages of the anisotropic simulation over the isotropic simulation. In addition, accurate predictions for the local failure of the additively manufactured component resulted from the anisotropic simulation. This study successfully developed a coupled process-structure simulation, considering the material orientation, to accurately predict mechanical behavior.

Through true stress-strain curve comparisons between simulations and experimental tensile tests, it was evident that the anisotropic and elastoplastic simulation presented a more realistic representation of the material behavior compared to isotropic simulations. The simulations not only provided accurate stress distributions but also replicated fracture behavior with remarkable precision.

Furthermore, the ability to model and predict the mechanical response for different strand orientations using a single, anisotropic and elastoplastic material model showcases the versatility and potential of the used simulation methodology. It allows for better understanding and prediction of material behavior under different loading conditions, enabling more informed design decisions and improvements in additive manufacturing processes. Other established materials that are processed using FFF processes, such as ABS or PETG, have similar anisotropies and plastic deformation behavior. This motivates the use of the approach presented here to also examine the materials and components manufactured with them.

In conclusion, this research lays the foundation for enhanced computational modeling and simulation techniques in the field of additive manufacturing, providing a valuable tool for engineers and researchers to optimize designs, predict material properties, and advance the application of 3D printing in diverse industries. Further testing and validation of this simulation approach will contribute to the ongoing progress and adoption of additive manufacturing technologies.

## Figures and Tables

**Table 1:** Tsai–Hill Parameter.

Tsai—Hill Parameters [Mpa]		
X	Y	S
49	31	15

**Table 2:** Material properties for PLA and inclusion.

Porosity		6.89	[%]
Parameters of inclusion			
Youngs modulus	E_f_	10	N/mm^2^
Possion’s ratio	n_f_	0.45	
Density	r_f_	1 × 10^−3^	g/cm^3^
Parameters of solid PLA			
Youngs modulus	E_m_	2700	N/mm^2^
Possion’s ratio	n_m_	0.35	[-]
Density	r_m_	1.3	g/cm^3^

**Table 3:** Average deviation of true stress over the course of elongation.

Strand Orientation	$\mathrm{Average}\;\mathrm{Deviation}\;\mathrm{of}\;\sigma_{w}$
0°	2.4%
45°	5.4%
90°	3.2%

**Table 4:** Comparison of fracture stress of tensile test and simulation.

Strand Orientation	$\sigma_{BT}$ Tensile Test	Standard Variance	$\sigma_{BS}$ Simulation	Deviation
0°	48.17 MPa	2.4 MPa	40.19 MPa	16.57%
45°	27.64 MPa	4.2 MPa	29.30 MPa	6.01%
90°	24.10 MPa	1.8 MPa	24.50 MPa	1.66%

**Table 5:** Comparison of elongation at break of tensile test and simulation.

Strand Orientation	$\varepsilon_{BT}$ Tensile Test	Standard Variance	$\varepsilon_{BS}$ Simulation
0°	0.0405	0.0008	0.0338
45°	0.0254	0.00023	0.0226
90°	0.0202	0.00039	0.0189

## Data Availability

The data presented in this study are available on request from the corresponding author.
